# Management of Chilli Anthracnose Using *Garcinia atroviridis* Nanoemulsions Integrated with *Trichoderma harzianum*

**DOI:** 10.3390/plants15081214

**Published:** 2026-04-15

**Authors:** Yasmeen Siddiqui

**Affiliations:** Department of Biological Sciences, College of Science, King Faisal University, Al Ahsa 31982, Saudi Arabia; ysiddiqui@kfu.edu.sa

**Keywords:** anthracnose, biocontrol, chilli, *Colletotrichum capsici*, *Garcinia atroviridis*, nanoemulsion, *Trichoderma harzianum*, yield

## Abstract

Chilli is a major horticultural crop in tropical and subtropical regions that contributes substantially to the global culinary and economic sectors. However, anthracnose remains one of the most destructive diseases, causing severe losses in both field and stored fruits. Current management strategies offer limited long-term effectiveness, highlighting the need for sustainable alternatives. This study developed nanoemulsions (NEs) from *Garcinia atroviridis* fruit extract and evaluated their biocontrol potential against *Colletotrichum capsici* alone and in combination with *Trichoderma harzianum*. Two formulations, NE4 and NE7, exhibited good thermostability without phase separation at 25 and 54 °C, with droplet sizes of 135.1 and 124.1 nm, respectively, and were non-phytotoxic to chilli seedlings. In vitro, the nanoemulsions significantly suppressed *C. capsici* mycelial growth (62%) compared to the crude extract. Under rain shelter conditions, NE integrated with *T. harzianum* (T7 and T8) was highly effective in delaying disease onset and reducing disease severity, achieving 90.07% and 88.37% relative disease reduction, respectively. These treatments also produced the highest marketable yields, comparable to the synthetic fungicide Dithane M-45^®^ (2 g L^−1^). In contrast, the untreated control group exhibited an 83% yield loss. The results indicate that nanoemulsions of *G. atroviridis* fruit extract, particularly when combined with *T. harzianum*, offer a promising and sustainable biological control option for managing pre-harvest chilli anthracnose. Their incorporation into integrated pest management programmes may reduce dependence on chemical fungicides and support safer chilli production systems.

## 1. Introduction

Chilli (*Capsicum annuum* L.; Solanaceae) is a vital horticultural crop cultivated worldwide for its economic, nutritional, and medicinal value. Plant pathogens cause major global crop losses [1], and post-harvest decay further exacerbates yield decline [2], thereby threatening food security. Overall, pests and diseases are responsible for an estimated 20–40% drop in global crop production, amounting to approximately US$220 billion in annual losses [3].

Anthracnose, mainly caused by *Colletotrichum* spp., affects fresh produce worldwide [4,5,6]. This is especially problematic in tropical and subtropical areas, where chilli yield losses of 10–80% have been reported [7,8]. The disease primarily affects mature fruits, causing significant decay before and after harvest [9,10,11].

Although fungicides such as strobilurins, pyraclostrobin, and azoles are commonly used to control the disease [11], excessive use has led to fungicide resistance, environmental contamination, and health concerns [12,13,14]. Thus, reducing reliance on synthetic fungicides and shifting toward biodegradable, eco-friendly alternatives is imperative.

Biocontrol agents and natural compounds offer promising low-impact disease management options [15,16,17,18]. Biological control agents (BCAs), including *Trichoderma*, *Bacillus*, and *Pseudomonas* spp., offer promising disease suppression through competitive exclusion, production of antifungal metabolites, and induction of plant defences. *Trichoderma* spp. has been reported to be an effective antagonistic biocontrol agent against post-harvest anthracnose in chilli. Moreover, another eco-friendly and efficient approach for managing chilli anthracnose involves the combined application of plant extracts from neem (*Azadirachta indica*), mahogany (*Swietenia mahagoni*), and garlic (*Allium sativum*). Combined extracts from these plants have significantly reduced disease and enhanced chilli yield [19]. The fungitoxic efficacy of phytoextracts varies according to their bioactive components, instability, and bioavailability.

Recent advancements have highlighted the potential of nanoemulsion-based formulations of plant extracts to enhance antimicrobial activity, stability, and bioavailability, while minimizing toxicity. The combination of nanoemulsions and BCAs may provide synergistic and durable protection against fungal pathogens.

Plant-derived bioactive compounds, particularly underutilized species, are gaining attention as sustainable pest management tools. *G. atroviridis*, traditionally used for its medicinal and preservative properties, contains diverse phytochemicals with antimicrobial potential [20]. However, its application in plant disease management remains largely unexplored. This study aimed to develop nanoemulsions of *G. atroviridis* fruit extract to evaluate their efficacy both alone and in combination with *T. harzianum* for chilli anthracnose management and yield improvement. The findings could contribute to the development of an integrated, environmentally friendly management strategy.

## 2. Results

### 2.1. Antifungal Activity

**Figure 1:**
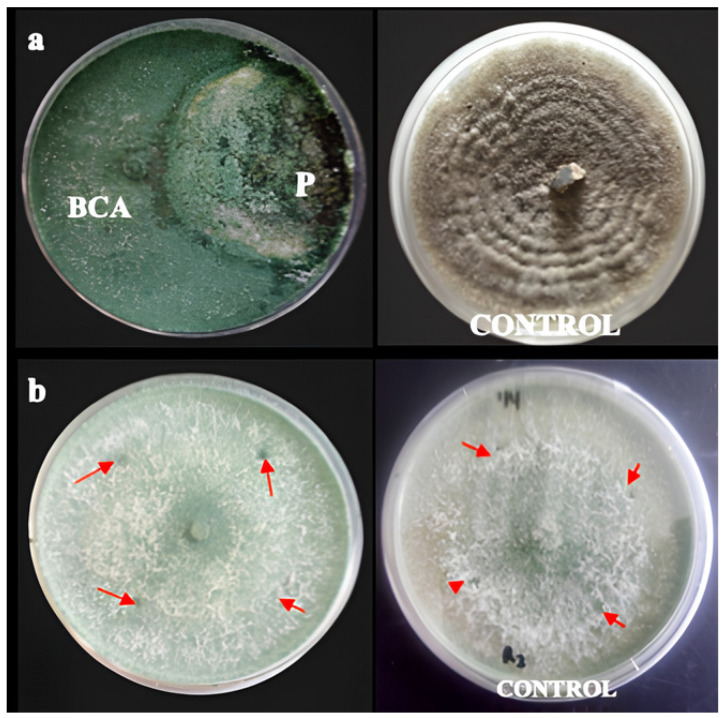
(**a**) Antagonistic activity of *Trichoderma harzianum* against (P) pathogen *Colletotrichum capsici* using a dual culture test in comparison to control. (**b**) Compatibility of *T. harzianum* in the presence of 100% of *G. atroviridis* fruit extracts in a 6 mm agar well (indicated by arrow); in comparison to the control plate.

The dual-culture assay showed that *T. harzianum* effectively suppressed the mycelial growth of *C. capsici*, achieving an approximately 75% inhibition after seven days. As incubation progressed, *T. harzianum* rapidly overgrew the plate surface and invaded the *C. capsici* colony, eventually dominating it (Figure 1a). When transferred to fresh PDA, only *T. harzianum* grew, indicating complete suppression of *C. capsici*. The in vitro compatibility test further showed that the *Garcinia* crude extract was non-inhibitory to *T. harzianum*, as no inhibition zones were observed around the wells containing the extract (Figure 1b).

### 2.2. Development and Physiochemical Characterization of Nanoemulsions

The NEs were prepared via spontaneous emulsification and aqueous titration. A ternary phase diagram (Figure S1) depicts two regions with varying percentages of mixtures: isotropic (L), which suggests a monophasic transparent/translucent liquid. Monophasic zones were identified at the apex line of deionised water and Tween 80 at 10 and 100% (*w*/*w*), respectively. Multiphase (MP) indicates biphasic or triphasic transparent or cloudy liquids. The multiphase region occupied most of the Tween 80 and oil–water apex lines, indicating instability and incompatibility in the emulsion system. The pre-formulation phase is an essential part of NE development because it determines the physicochemical properties and stability of the nanoemulsion. Seven pre-formulations (PF1, PF2, PF3, PF4, PF5, PF6, and PF7) were selected from the monophasic domain of the phase diagram for pre-formulation screening (Table S1).

#### 2.2.1. Thermostability and Stability

Environmental factors significantly influence emulsion stability. The results indicated that the pre-formulations were generally stable after 60 days of storage at room temperature. Centrifugation did not cause cracking or phase separation. The thermostability during pre-formulation storage at 28, 54, and 4 °C is tabulated (Table S2). PF1, PF2, PF4, PF5, PF6, and PF7 did not exhibit phase separation at room temperature (25 °C) for 60 days. At 54 °C, after 14 d of storage, PF2 and PF5 showed phase separation with creaming and were transformed into two opaque phases. In contrast, all pre-formulations were unstable at 4 °C. Pre-formulations PF1, PF3, and PF5 formed two phases, whereas PF2, PF4, PF6, and PF7 formed three phases. Therefore, these formulations are unsuitable for storage at low temperatures. The formulations that survived the thermostability test were selected for the development of nanoemulsions and further characterization.

#### 2.2.2. Surface Tension, Polydispersity Index, Droplet Size and Zeta Potential

The selected formulations were subjected to progressive aqueous dilution with gentle stirring to generate NE1, NE2, NE3, NE4, NE5, NE6, and NE7. Table 1 presents the characterization of various NE formulations optimized using a ternary phase diagram. The development of nanoemulsions is a non-spontaneous and kinetically steady process that requires external energy to induce dispersion. Surface tension plays an important role in influencing liquid fluidity and determining droplet formulation. All NE formulations had surface tensions ranging from 29.5 to 30.5 mN/m. Emulsions with droplet sizes less than 200 nm were considered nano-sized, whereas those larger than 200 nm were designated micron-sized. Except for NE5 (205 nm), all formulations had small mean droplet sizes ranging from 124.1 to 176 nm, along with low PDI values between 0.157 and 0.422. In general, all formulations had negative zeta potentials. However, NE4 and NE7 had charges greater than −30 mV, suggesting that the nanoemulsion formulations were relatively stable.

### 2.3. Antagonistic Activity of Nanoemulsions

Under in vitro conditions, all nanoemulsions of *G. atroviridis* fruit extracts inhibited the development of *C. capsici* (Table 2). The zones of inhibition produced by the nanoemulsions were insignificant in comparison with the commercially available chemical control (Dithane-45^®^). However, the fungicide (Dithane-45^®^) and the NE2, NE3 formulations demonstrated the largest zones of inhibition, followed by NE5, NE7, NE4, NE1 and NE6 in descending order of inhibition zones. Compared with crude extracts, the nanoemulsion formulation had a higher PIRG score.

### 2.4. Phytotoxicity

Only two nanoemulsions, NE4 and NE7, were selected and used in the rain shelter study. Both NE formulations yielded the best results, with no observed phytotoxicity. The efficacy of the NE formulations was compared with that of the water-sprayed control plants. The results revealed that NE2, NE3, NE5, and NE6 caused phytotoxic effects, including chlorosis of young leaves, followed by marginal scorching and reduction in the size of newly emerged leaves, eventually resulting in leaf fall and causing complete necrosis of the treated plant. The phytotoxic effect was evident two days after spray application. The burnt leaf margins gradually became necrotic, leading to mortality after 30 d (NE2 and NE3), whereas the control plants remained healthy.

### 2.5. Disease Incidence and Severity

#### 2.5.1. Disease Development in Chilli Plants

**Figure 2:**
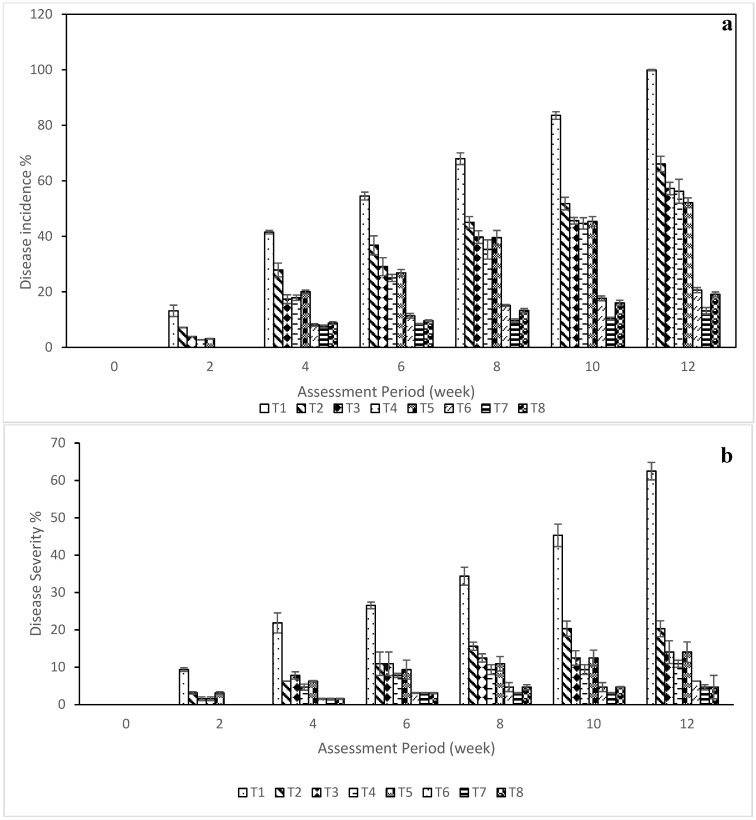
(**a**) Disease incidence and (**b**) disease severity assessed in chilli leaves infected by *C. capsici* treated with different nano formulations alone or integrated with *T. harzianum*. Each value represents the mean of four replicates. The vertical bar indicates standard deviation. T1 = water (control); T2 = crude extract of *Garcinia atroviridis*; T3 = nanoemulsion of crude extract (NE4); T4 = nanoemulsion of crude extract (NE7); T5 = aqueous suspension of *T. harzianum*; T6 = Dithane M-45^®^ @ 2 g L^−1^; T7 = nanoemulsion of crude extract (NE4) + *T. harzianum*; T8 = nanoemulsion of crude extract (NE7) + *T. harzianum*.

Disease incidence based on foliar symptoms is shown in Figure 2a. No visual symptoms were observed immediately after inoculation. Anthracnose development, characterized by circular necrotic and chlorotic lesions on stems and leaves, increased progressively at 2, 4, 6, 8, 10, and 12 WAI (Figure S2). A significant treatment effect was observed (*p* < 0.001). At 12 WAI, the lowest disease incidence was recorded in plants treated with the NE of crude extract (NE4) + *T. harzianum* (T7, 13.1%), followed by the NE (NE7) + *T. harzianum* (T8), Dithane M-45^®^ (T6), aqueous *T. harzianum* (T5), NE7 alone (T4), and NE4 alone (T3). The highest incidence occurred with crude *G. atroviridis* extract (T2, 66.08%) and the water control (T1, 99.89%). No symptoms were observed in the uninoculated control (T9).

Disease severity varied significantly among treatments (Figure 2b). The water control (T1) exhibited the highest disease severity, with a disease index of 4 (62.5%). In contrast, plants treated with the NE crude extract + *T. harzianum* (T7 and T8) showed the lowest severity (4.69%, disease index = 1), resulting in 88–90% disease reduction. Dithane M-45^®^ (T6), NE alone (T4 and T3), aqueous *T. harzianum* (T5), and crude *G. atroviridis* extract (T2) showed intermediate severity levels (6.25–20.31%). Mild phytotoxic symptoms, including marginal leaf scorching, were observed in fungicide-treated plants, whereas water-soaked lesions and chlorosis occurred in crude-extract treatments.

ANOVA revealed highly significant treatment effects on disease progression parameters [F(8,18) = 1778.20, *p* < 0.001]. Tukey’s post hoc test indicated that the untreated control (T1) showed the highest epidemic rate (rL = 1.80), whereas T6–T8 significantly reduced disease progression (rL = 0.786–0.500). Treatments T2–T5 provided moderate suppression. Cumulative disease development (AUDPC) differed significantly among treatments [F(8,18) = 8877.94, *p* < 0.001]. The control recorded the highest AUDPC value (276.6), while T7, T8, and T6 showed the lowest values (30–33), indicating delayed and suppressed disease development (Table 3). Disease reduction also varied significantly [F(8,18) = 3002.54, *p* < 0.001]. No reduction was observed in T1, whereas T6–T8 achieved an 88–90% reduction. Moderate disease reduction (62–72%) was observed in T2–T5.

#### 2.5.2. Disease Development in Chilli Fruit

**Figure 3:**
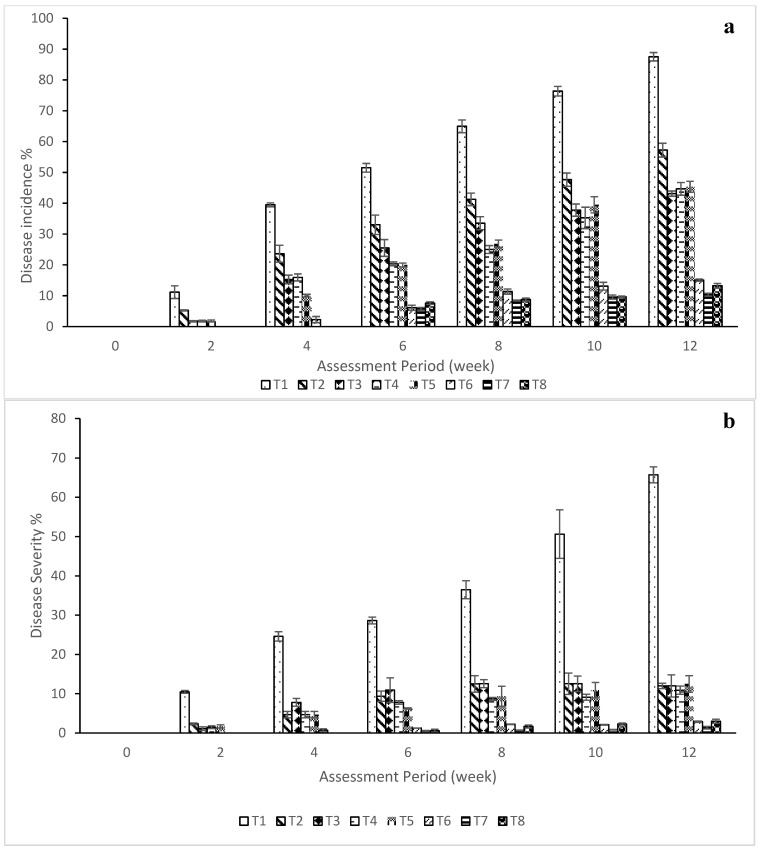
(**a**) Disease incidence and (**b**) disease severity assessed in chilli fruits infected by *C. capsici* treated with different nano formulations alone or in combination with *T. harzianum*. Each value represents the mean of four replicates. The vertical bar indicates standard deviation. T1 = water (control); T2 = crude extract of *Garcinia atroviridis*; T3 = nanoemulsion of crude extract (NE4); T4 = nanoemulsion of crude extract (NE7); T5 = aqueous suspension of *T. harzianum*; T6 = Dithane^®^ M-45 @ 2 g L^−1^; T7 = nanoemulsion of crude extract (NE4) + *T. harzianum*; T8 = nanoemulsion of crude extract (NE7) + *T. harzianum*.

Disease incidence caused by *C. capsici* was assessed every 2 WAI at fruit set based on the appearance of sunken circular or angular lesions with concentric acervuli. Significant differences were observed among treatments. The water-treated control (T1) recorded the highest fruit infection (87.54%), whereas the NE of crude extract (NE4) + *T. harzianum* (T7) showed the lowest incidence (10.35%), followed by T8, T6, T3, T4, T5, and T2, with values of 13.24%, 15.08%, 43.13%, 44.68%, 45.33%, 57.24%, and 87.54%, respectively, at 12 WAI (Figure 3a). No symptoms were observed in the non-inoculated plants (T9). Disease severity followed a similar trend (Figure 3b), with T7 and T8 showing the lowest severity index (1), followed by T6.

ANOVA revealed highly significant treatment effects on the epidemic rate (rL) (F(8,18) = 1032.93, *p* < 0.001), AUDPC [F(8,18) = 479.14, *p* < 0.001], and disease reduction (F(8,18) = 469.02, *p* < 0.001), confirming differential treatment efficacy against fruit anthracnose.

The mean rL values ranged from 0.00 to 0.95 (Table 4). T1 showed the highest epidemic rate (0.95 ± 0.07), followed by T2, T4, T3, T5, T6, T8, and T7 (0.868–0.531). Treatments T6–T8 significantly reduced rL compared with the control but did not differ from each other (*p* > 0.05). Tukey’s HSD grouped T7, T8, and T6 into the most effective category, with T7 exhibiting the lowest disease progression.

AUDPC patterns corresponded with rL values. The highest AUDPC was recorded in T1 (342 units), whereas T7 and T8 showed the lowest values (26.56 and 32.81, respectively). Treatments T6–T8 significantly slowed disease development and prolonged symptom-free periods, indicating effective suppression of lesion expansion and cumulative disease intensity.

Disease reduction also differed significantly among the treatments (*p* < 0.05). T7 exhibited the highest reduction (91.86%), followed by T8 and T6 (68–72%). These three treatments were statistically similar (*p* > 0.05) but significantly more effective than T1–T5, with mean differences of +23.82 to +100%. Overall, treatments T6–T8 provided the strongest protection, with T7 consistently outperforming all other treatments.

### 2.6. Marketable Yield and Yield Loss

The integrated application of a nanoemulsion (NE) crude extract with *T. harzianum* significantly affected anthracnose severity, fruit number, yield, and yield loss in artificially inoculated chilli plants. One-way ANOVA revealed significant treatment effects on all measured parameters (*p* ≤ 0.05).

Plants treated with Dithane^®^ M-45 and the NE crude extract + *T. harzianum* (T7 and T8) produced healthy, bright-red, lesion-free fruits comparable to healthy uninoculated chilli plants. In contrast, fruits from plants treated with crude *G. atroviridis* extract alone (T2) showed mild blemishes, whereas the inoculated water-sprayed control (T1) exhibited severe anthracnose symptoms.

**Figure 4:**
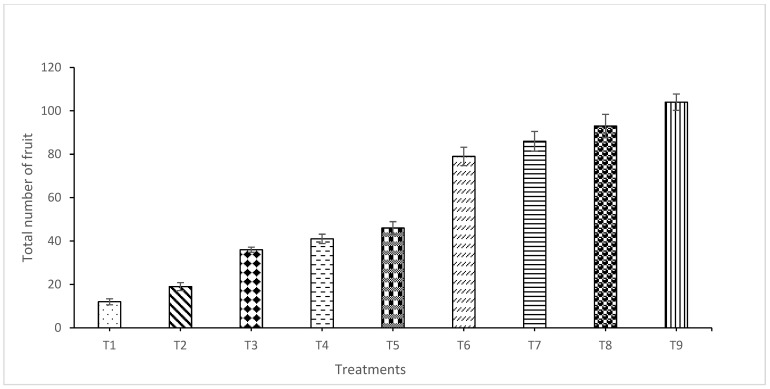
Effect of nano formulations alone or integrated with *T. harzianum* on the total number of chilli fruits per plot. Each value represents the mean of four replicates. The bars indicate standard deviation. T1 = water (control); T2 = crude extract of *Garcinia atroviridis*; T3 = nanoemulsion of crude extract (NE4); T4 = nanoemulsion of crude extract (NE7); T5 = aqueous suspension of *T. harzianum*; T6 = Dithane M-45^®^ @ 2 g L^−1^; T7 = nanoemulsion of crude extract (NE4) + *T. harzianum*; T8 = nanoemulsion of crude extract (NE7) + *T. harzianum*; T9 = healthy.

**Figure 5:**
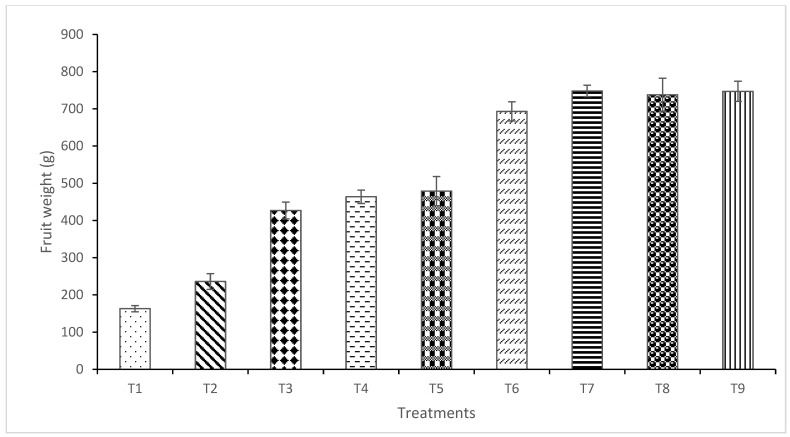
Effect of nano formulations alone or integrated with *T. harzianum* on yield of chilli fruits per plot. Each value represents the mean of four replicates. Vertical bars indicate standard deviation. T1 = water (control); T2 = crude extract of *Garcinia atroviridis*; T3 = nanoemulsion of crude extract (NE4); T4 = nanoemulsion of crude extract (NE7); T5 = aqueous suspension of *T. harzianum*; T6 = Dithane M-45^®^ @ 2 g L^−1^; T7 = nanoemulsion of crude extract (NE4) + *T. harzianum*; T8 = nanoemulsion of crude extract (NE7) + *T. harzianum*; T9 = healthy.

**Figure 6:**
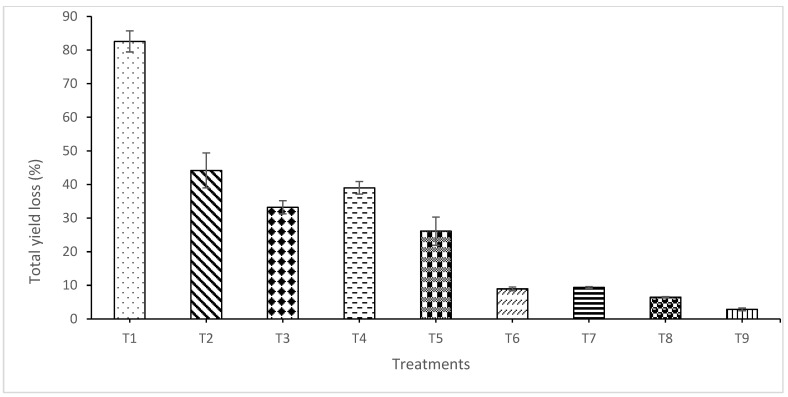
Total chilli yield loss per plot treated with different nano formulations alone or integrated with *T. harzianum*. Each value represents the mean of four replicates. Vertical bars indicate standard deviation. T1 = water (control); T2 = crude extract of *Garcinia atroviridis*; T3 = nanoemulsion of crude extract (NE4); T4 = nanoemulsion of crude extract (NE7); T5 = aqueous suspension of *T. harzianum*; T6 = Dithane M-45^®^ @ 2 g L^−1^; T7 = nanoemulsion of crude extract (NE4) + *T. harzianum*; T8 = nanoemulsion of crude extract (NE7) + *T. harzianum*; T9 = healthy.

Results indicated a significant difference in fruit number among treatments (*p* ≤ 0.05) (Figure 4). The highest number of fruits was recorded for T7 (86 fruits/plot) and T8 (93 fruits/plot), whereas T1 recorded the lowest fruit number. The reduced fruit set in T1 was associated with the establishment of pathogens early during the flowering stage. Similarly, significant variations in the total fruit yield were observed among treatments (*p* ≤ 0.05). The highest yield was recorded in T7 (748 g/plot), followed by T8 (738 g), T6 (693 g), T5 (479 g), T4 (464 g), T3 (427 g), and T2 (236 g) (Figure 5). The lowest yield (163 g/plot) was recorded for the water-treated control (T1). Yield loss differed significantly across treatments, with the highest loss (82.58%) observed in T1 (Figure 6). These results demonstrate that the combined application of the NE crude extract with *T. harzianum* (T7 and T8) effectively suppressed anthracnose and significantly enhanced fruit yield in chilli.

## 3. Discussion

*Trichoderma* spp. is widely recognized as a dominant soil fungus and an effective biocontrol agent, functioning through both direct and indirect interactions in the phyllosphere. Its antagonistic activity against *Colletotrichum* sp. involves rapid colonization, competition for nutrients, and mycoparasitism, leading to coiling and parallel growth of pathogen hyphae [21,22]. In the present study, *T. harzianum* inhibited the mycelial growth of *C. capsici* by up to 75%. Similar findings were reported by De la Cruz-Quiroz et al. [23] for *C. gloeosporioides*. *Trichoderma* spp. secretes hydrolytic enzymes, such as chitinases, that degrade the glycosidic linkages in fungal cell walls, leading to hyphal penetration and host death [24,25,26]. Loc et al. [27] reported that chitinase from *T. asperellum* PQ34 at 60 U mL^−1^ almost completely inhibited *Colletotrichum* growth in vitro, supporting the ability of *Trichoderma* to employ multiple biocontrol mechanisms.

Extracts of *G. atroviridis* have demonstrated antimicrobial activity against a range of microorganisms [20]. Mokhtar et al. [28] showed that *G. atroviridis* fruit extract inhibited *C. capsici* by 70.5% using the agar plate method. Based on these findings, a combination of *G. atroviridis* and *T. harzianum* was selected to develop an integrated management approach against chilli anthracnose. Compatibility studies revealed that *T. harzianum* was not inhibited by the crude *G. atroviridis* extract. Similar observations were reported by Vanitha [29], who found that the phytoextracts of wintergreen and lemongrass oils were compatible with *Trichoderma* spp. under in vitro conditions. Tapwal et al. [30] further reported that aqueous extracts of *Parthenium hysterophorus*, *Urtica dioeca*, and *Adiantum venustum* were fully compatible with *T. viride*, although reduced compatibility was observed with *Polystichum squarrosum* and *Cannabis sativa* at higher concentrations.

Emulsions are biphasic systems that are widely applied in the pharmaceutical and agricultural fields as delivery systems for macromolecules. Emulsion stability is critical for effective formulation performance. Therefore, pseudoternary phase diagrams were used to identify stable emulsion regions. Tween 80 was selected as the surfactant because of its effectiveness as an oil-in-water (O/W) emulsifier, which improves the affinity of hydrophobic compounds for aqueous phases by reducing surface tension [31]. Tween 80 rapidly adsorbs onto droplet surfaces and produces smaller droplet diameters than polymer surfactants.

The L regions in phase diagrams represent transparent, thermodynamically stable nanoemulsions with smaller droplet sizes, unlike the MP regions, which are prone to phase separation over time. Oil–water emulsions are inherently unstable and require surfactants for stabilization [32]. In the present study, centrifugation (3000–6000 rpm for 1.5 h) was applied to accelerate physical instability processes, such as creaming and coalescence [33,34]. The formulated nanoemulsions were unstable at 4 °C but stable at 25 °C and 54 °C, likely due to oil droplet expulsion and membrane rupture at lower temperatures [31]. Therefore, refrigeration is not recommended for storage. The formulation should be maintained under moderate temperature conditions to preserve stability and efficacy. These findings reinforce the importance of ingredient composition and ratios in achieving stable nanoemulsions [35,36]. Future work should focus on optimizing formulation components to improve low-temperature stability and shelf life. However, not every combination of components tested produced nanoemulsions, and the findings are in line with those of Shakeel et al. [37], who reported that none of the combinations in the entire range of possible compositions produced nanoemulsions.

The nanoemulsions prepared in this study showed low surface tension, which is critical for enhanced spreading and absorption [38,39]. Increasing the surfactant concentration significantly reduces the surface tension and droplet size, producing transparent solutions with improved oil–water interfaces [40,41,42]. The nanoemulsions exhibited droplet sizes below 200 nm, negative zeta potential, and high kinetic stability. The zeta potential reflects the surface electrical charge of droplets and is a key indicator of formulation stability [43]. Negative zeta potential values promote electrostatic repulsion, thus preventing droplet aggregation [44]. The small droplet size further enhances emulsion stability by minimizing flocculation and maintaining transparency [45,46]. These properties suggest that the developed nanoemulsions are suitable for spray applications in chilli anthracnose management.

Phytotoxicity evaluation revealed mild to severe toxicity in some nanoemulsion treatments, which was expressed as chlorosis, necrosis, or seedling damage. Allelopathic compounds in plant tissues may be released under specific conditions and exhibit phytotoxic properties [47]. Although essential oils (EOs) are generally regarded as safe (GRAS) by the FDA and EPA, their phytotoxic effects have been reported in some cases [48,49]. Surfactants used in nanodispersions may also contribute to phytotoxicity. Treatments NE1, NE2, NE3, NE5, and NE6 showed higher toxicity, likely due to elevated surfactant levels. Surfactants can enhance cuticular permeability, biocidal efficacy, and phytotoxicity through synergistic interactions [50,51]. Excessive surfactant concentrations also reduce chlorophyll fluorescence and impair photosynthesis [52]. In contrast, NE4 and NE7 exhibited minimal phytotoxicity, which may be attributed to their relatively balanced oil-to-surfactant ratios and lower effective surfactant concentrations. These formulations also demonstrated improved physicochemical stability, which may have contributed to the controlled release and reduced phytotoxic effects.

All nanoemulsions exhibited significantly greater antifungal activity against *C. capsici* than crude extracts. The activity of plant extracts is attributed to secondary metabolites, including terpenoids, phenols, flavonoids, and alkaloids [53,54,55]. Moreover, the physicochemical characteristics likely contributed to the enhanced biological activity of the nanoemulsions. Smaller droplet sizes provide a larger surface area for interaction with fungal cells, improving the delivery and penetration of bioactive compounds present in the fruit extracts. In addition, stable nanosized droplets with sufficient electrostatic repulsion remain well-dispersed in aqueous media, facilitating uniform deposition on plant surfaces during spray application.

Several studies have reported disease suppression using combined phytoextracts and biocontrol agents [56,57,58]; however, research integrating nanoemulsions with biological agents remains limited. *Trichoderma* spp. are ideal biocontrol agents because of their rapid growth, antibiotic production, mycoparasitic activity, and adaptability to different substrates [59,60]. They suppress pathogens through spatial competition and secondary metabolite production [61,62]. The combination of *Trichoderma* with plant extracts, such as onion, garlic, neem, and acacia, has shown significant antifungal activity against *C. capsici*, while improving chilli quality [56].

Anthracnose is a major pre- and post-harvest disease of chilli, causing significant economic losses. In this study, nanoemulsion treatments T3 and T4 significantly reduced fruit anthracnose (67.88% and 76.18%, respectively) compared with the crude extract (61.14%). Integrated treatments (T7 and T8) further enhanced disease control, delaying disease onset by four weeks and achieving up to a 90% disease reduction, whereas independent treatments provided only a 60–70% suppression. These findings are in line with those which demonstrate that nanotechnology-based biopesticides, such as neem oil nanoemulsions, essential oil nanodispersions, and chitosan nano formulations, achieve similar levels of disease suppression in various crops, often ranging from 80 to 92% under controlled and field conditions (Mondel et al. [63]). The enhanced efficacy of nanoemulsions is attributed to improved wettability, uniform deposition, and enhanced membrane permeability [64,65], leading to reduced minimum inhibitory concentrations and improved fungicidal performance compared with crude extracts. Similar findings were reported using clove and black seed oil nanoemulsions against *Botrytis cinerea* Ziedan et al. [66].

Anthracnose symptoms include sunken lesions with concentric acervuli producing pink to orange conidial masses [2,67]. Quiescent infections on immature fruits become active during ripening, drastically reducing fruit quality. Even minor lesions render fruits unmarketable [68]. The market value is strongly influenced by fruit appearance, moisture content, and mass [69]. Yield assessment demonstrated that the disease incubation period strongly affected yield loss. Integrated nanoemulsion *T. harzianum* treatments (T7 and T8) significantly reduced yield loss and enhanced marketable yield compared to the control. This confirms that integrated nanobiological formulations effectively suppress *C. capsici* under both in vitro and in vivo conditions, resulting in improved fruit quality and yield.

## 4. Materials and Methods

Emereen^TM^ 2126 palm kernel oil (HLB:14) was purchased from Emery Oleochemical, Sdn. Bhd., Selangor, Malaysia and Tween 80 (HLB:15) was purchased from R&M Chemicals, Malaysia. Unprocessed, air-dried, and sliced raw *G. atroviridis* fruits were obtained from LSK Fishery Sdn. Bhd., Penang, Malaysia. *T. harzianum* isolate UPM40 was obtained from the microbial culture collection unit at the Institute of Bioscience, Universiti Putra Malaysia, Selangor, Malaysia, whereas *C. capsici* (accession no. GU227862) was isolated from anthracnose-infected chilli [28].

### 4.1. Antagonistic Activity

Pure *T. harzianum* and *C. capsici* cultures were prepared and maintained on potato dextrose agar (Difco^TM^ PDA). A 5 mm diameter agar plug taken from a 7-day-old culture of *C. capsici* was placed 2 cm away from the periphery of a 9 cm diameter Petri plate containing PDA. Another agar plug of the same size and age as that of *T*. *harzianum* was placed 5 cm away from the *C. capsici* plug and incubated at 28 ± 2 °C until the control plate was fully covered by *C. capsici* mycelium. The percentage inhibition was calculated with reference to the control.

### 4.2. Compatibility Study

Crude *Garcinia* extract was prepared following Mokhtar et al. [28]. Briefly, sun-dried fruit samples (33 °C, 48 h; 300 g) were ground, and the resulting powder (245 g) was extracted in 1 L methanol for 72 h on a shaker, then left to settle for 24 h. The mixture was filtered (Whatman No. 1), and the filtrate was concentrated to one-fifth of its volume using a rotary evaporator. The extract was adjusted to 900 μg mL^−1^ with sterile distilled water. Agar well diffusion plates were prepared by pouring 15 mL of molten PDA into each Petri dish. After solidification, four 6 mm wells were cut into the agar. A 5 mm plug of *T. harzianum* from a 7-day-old culture was placed at the centre of each plate. Next, 10 μL of crude extract was pipetted into each well. Sterile distilled water (10 μL) served as the control. All plates were incubated at 24 ± 2 °C for seven days, and the inhibition zones (cm) were measured.

### 4.3. Nanoemulsion Preparation

A pseudoternary phase diagram was constructed to identify the optimal component ratios for nanoemulsion using Emereen™ 2126 oil, Tween 80, and water, following the aqueous titration method of Shaaban and Edris [70]. Oil and surfactant were mixed in glass vials at weight ratios ranging from 10:0 to 0:10 (*w*/*w*), and 1 mL of *Garcinia* fruit extract was added to each mixture. Water was then titrated incrementally from 5% to 95% (*w*/*w*). Each mixture was vortexed (VTX-3000L, Tokyo, Japan), centrifuged at 3500 rpm for 30 min at 25 °C, and then examined under crossed polarizers to determine phase behaviour. The phase diagram was generated using Chemix School version 3.5 (Bergen, Norway), distinguishing isotropic (transparent, single-phase) from anisotropic (cloudy, two-phase) regions. Pre-formulations were selected based on optical isotropy and stability at room temperature and after centrifugation at 6000 rpm for 1 h (IKA G-L, IKA-Werke GmbH & Co., Staufen, Germany). Water was added dropwise to stable pre-formulations and stirred at 200 rpm for 5 min using an IKA RW20 digital overhead stirrer (IKA-Werke GmbH & Co., Staufen, Germany) until the nanoemulsions formed. An approximate water-to-pre-formulation ratio of 1:200 was required.

#### 4.3.1. Stability and Thermostability

The stability and thermostability of the selected pre-formulations were tested by centrifugation at 3500 rpm (Eppendorf, Hamburg, Germany) for 30 min and incubation at room temperature at 28 °C for 2 months, and 54 °C and 4 °C for 2 weeks, as a standard evaluation for agrochemical products to show stability in a tropical climate, as prescribed by the FAO [71]. This step was repeated twice.

#### 4.3.2. Surface Tension, Droplet-Size Distribution, Zeta Potential and Polydispersity Index

The surface tension of formulations was measured on a Kruss K6 tensiometer equipped with a platinum plate (Kruss, Bristol, UK) based on the Du Nouy ring immersion technique, performed at 25 °C. Before measurement, calibration was conducted using deionised water with a surface tension of 72–73 mN/m, and a Zetasizer Nano-ZS particle size analyser (Malvern Instruments, Malvern, UK) with a laser reader of 633 nm was used to determine the droplet size and zeta potential of the developed NEs. The electrical charge on the oil droplets in the emulsions was determined at an electrical voltage of 3.9 V. To examine the polydispersity index (PDI) of the samples, dynamic light scattering (DLS) was performed at a 90° angle at 25 °C using a Zetasizer NanoZS (Malvern Instruments, Malvern, UK). Three replicates were performed for each formulation.

### 4.4. Antagonistic Activity of Nanoemulsions

Agar well plates were prepared as described in Section 2.1. A 6 mm diameter plug of *C. capsici*, obtained from an actively growing region of a 7-day-old culture, was placed at the centre of the agar well plate. Subsequently, 10 μL of each nanoemulsion (NE1, NE2, NE3, NE4, NE5, NE6, and NE7) was dispensed into each well. Dithane M-45^®^ (2 g L^−1^) was used as a positive control. All plates were incubated at 24 ± 2 °C for 7 days. The appearance of clear halos around the wells indicated antifungal activity. The diameters (cm) of the inhibition zones were measured using a scale.

### 4.5. Phytotoxicity

The phytotoxicity of the selected nanoemulsions was assessed in 21-day-old chilli plants, with water serving as the control. Seeds of a local variety (HP855 GWG–Green World Genetic) were obtained from the Department of Agriculture, Serdang, Selangor, Malaysia. Seedlings were raised in peat moss and transplanted into 12 × 12 cm polybags containing the same medium after three weeks. The plants were manually watered. One week after establishment, each nanoemulsion (NE1-NE7) and the control were sprayed to runoff. Each treatment was performed in triplicate. Phytotoxicity was rated on a 0–5 scale based on foliage discoloration, chlorosis, and necrosis [0 = no symptoms; 1 = 1–25% very slight discoloration; 2 = 26–50%, moderate and lasting discoloration; 3 = 41–60%, moderately heavy necrotic symptoms; 4 = 61–80%, heavy necrosis followed by leaf fall to nearly destroyed seedling; and 5 = 81–100%, dead] that destroyed the seedling. Formulations exhibiting no or minimal phytotoxicity were selected for rain-shelter trials.

### 4.6. Integrated Effect of Nanoemulsions and T. harzianum on the Yield and Development of Chilli Anthracnose

Chilli seeds were germinated as described in Section 2.5. After three weeks, uniformly grown seedlings were transplanted into 20 × 20 cm polybags filled with 3 kg of coco peat. Recommended fertigation (modified standard copper formulation) was applied directly to the root zone (Table S3) according to the electrical conductivity of the planting medium at different growth stages. Artificial inoculation with *C. capsici* was conducted at the flowering stage (45 days after transplantation). The inoculum was prepared from a 20-day-old culture on PDA, and the spore concentration was adjusted to 1 × 10^6^ spores mL^−1^ using a haemocytometer; 0.1 mL of Tween 20 per 250 mL suspension was added as a surfactant. Each plant was spray-inoculated with 250 mL of the spore suspension and covered with a transparent plastic bag for 24 h. An aqueous suspension of *T. harzianum* was prepared from a 10-day-old culture, yielding 1 × 10^8^ CFU mL^−1^. The first spray treatment (Table 5) was applied two days after inoculation, followed by applications at 14-day intervals. Sprays were applied to the runoff using a handheld low-volume electric sprayer. The uninoculated plot served as the healthy control. Treatments were arranged in a randomized complete block design (RCBD) with four replicates, each containing 20 plants, and blocks spaced 2 m apart.

#### 4.6.1. Disease Incidence and Severity

Disease incidence (DI) was calculated based on the development of anthracnose symptoms on leaves and fruits per plant per plot and expressed as a percentage of the total number of plants observed [72].

For disease severity, the standard disease rating scale (0–5 scale) for assessing chilli anthracnose was modified from Montri et al. [73] as follows: 0 = no symptoms on leaf, branch, or fruit; 1= small, irregular brown spots covering 1% or less of an area of a leaf, branch, or fruit; 2 = brown, dirty, pin-headed spots covering 1–10% of the area on a leaf, branch, or fruit; 3 = dark brown, dirty black spots with a blackish margin covering 11–25% of the area of leaf, branch, or fruit; 4 = dark brown, circular, or irregular spots with blackish margins covering 26–50% of the area of a leaf, branch, or fruit; and 5 = dark brown, circular, or irregular spots with blackish margin covering 51% and above the area of a leaf, branch, or fruit. Disease severity was expressed as a percentage (DS %) and disease index (DI) as described by Campbell and Madden [74].

$$DS\%=\frac{\Sigma \;(Number\;of\;plants/fruits\;in\;that\;rating\times severity\;rating)}{Total\;number\;of\;seedlings/fruits\;assessed\times Highest\;scale}\times 100$$

The effectiveness of treatment in slowing disease progression was demonstrated by showing a decrease in disease severity, using the same data depicted in the disease progression curve. The slopes of the disease progression curve were obtained by transforming the disease severity data using the model (logit) of Campbell and Madden [74] and linear regression analyses using the Sigma Plot (version 9.0) software.

#### 4.6.2. Determination of Yield

The first harvest at the red colour stage (8 weeks after inoculation) was recorded for the entire trial. After harvest, the number and weight of the fruits per plot were counted and measured using a balance, respectively. The average total yield and yield of infected fruits were calculated for each plot (treatment). Marketable yield and yield loss were determined per plot using the following formula:

Marketable=Total yield−Infected fruit yield

$$Yield\;Loss(\%)=\frac{Total\;yield-Marketable\;yield}{Total\;yield}\times 100$$

### 4.7. Statistical Analysis

Statistical analyses were performed using the SAS statistical software (version 8.2; 2001; SAS Institute, Cary, NC, USA). Data were analyzed using analysis of variance (ANOVA), and means were compared using the post hoc Tukey HSD test.

## 5. Conclusions

The present study reveals that nanoemulsions derived from *Garcinia atroviridis* fruit extract offer a promising, environmentally sustainable method for managing chilli anthracnose. The selected nanoemulsion formulations exhibited favourable physicochemical stability, nanoscale droplet size, and no phytotoxic effects on chilli plants. Nevertheless, all pre-formulations were unstable at 4 °C, indicating a practical limitation for cold storage. Future research should aim to enhance low-temperature stability to facilitate storage and field application while effectively inhibiting the growth of *Colletotrichum capsici*. Notably, their combination with *Trichoderma harzianum* significantly delayed disease onset, reduced disease severity, and enhanced marketable yield under greenhouse conditions, with performance comparable to that of a commercial fungicide. These findings underscore the synergistic potential of botanical nanoemulsions and beneficial microbes as an effective biological control strategy. However, the scope of inference is limited to the protected experimental environment. Future studies should focus on validating the efficacy, stability and practical applicability of these formulations under field conditions and diverse agro-climatic environments to support their potential integration into sustainable disease management strategies for chilli production systems.

## Figures and Tables

**Table 1:** Polydispersity index, surface tension, droplet size and zeta potential of the nanoemulsion formulations prepared from selected pre-formulations.

Formulation	Formulation Composition (*w*/*w*)			Polydispersity Index	Surface Tension mN/m	Droplet Size (nm)	Zeta Potential (mV)
	S	O	W				
NE1	25	20	55	0.261 c ± 0.002	30.31 a ± 0.43	175.4 b ± 0.6	−10.9 e ± 0.9
NE2	20	40	40	0.422 a ± 0.003	29.51 b ± 0.10	156.1 c ± 1.2	−10.3 e ± 1.1
NE3	23	10	67	0.324 b ± 0.001	30.15 a ± 0.20	168.1 b ± 1.6	−15.2 d ± 1.9
NE4	20	30	50	0.226 bc ± 0.003	30.31 a ± 0.20	135.1 d ± 1.1	−31.5 a ± 1.2
NE5	15	35	50	0.238 bc ± 0.004	30.51 a ± 0.10	205.0 a ± 0.9	−18.9 c ± 1.7
NE6	15	30	55	0.227 bc ± 0.001	29.94 b ± 0.24	176.6 b ± 1.1	−25.6 b ± 1.4
NE7	5	5	90	0.157 d ± 0.002	30.21 a ± 0.10	124.1 d ± 0.9	−32.8 a ± 0.9

NE, nanoemulsion; S, surfactant; O, oil; and W, water. Means with the same letters within columns are not significantly different at *p* ≤ 0.05 according to the Tukey test. All data are presented as the mean ± SD (*n* = 4).

**Table 2:** Effect of different NE formulations of *G. atroviridis* fruit extract, crude extract and fungicide on the inhibition of *C. capsici* in agar well bioassay.

Formulation	Inhibition Zone (cm)
NE1	2.92 a ± 0.01
NE2	2.96 a ± 0.01
NE3	2.96 a ± 0.01
NE4	2.92 a ± 0.01
NE5	2.94 a ± 0.01
NE6	2.90 a ± 0.01
NE7	2.94 a ± 0.02
Crude extract	1.12 b ± 0.02
Dithane M-45^®^ @ 2 g L^−1^	2.95 a ± 0.03

Means followed by a common letter are not significantly different at *p* ≤ 0.05, according to the Tukey test, and are also presented as mean ± SD (*n* = 4).

**Table 3:** Disease progress rates, area under the disease progress curve (AUDPC), and percentage anthracnose disease reduction in chilli plants inoculated with *C. capsici* and treated with different nanoemulsions alone or in combination with *T. harzianum*.

Treatment	Disease Progress Rate (Units/Week)	AUDPC (Unit Square)	(%) Reduction
T1	1.854 a ± 0.051	271.02 a ± 5.55	0 c ± 0.00
T2	0.860 ab ± 0.007	106.53 b ± 1.84	62.30 b ± 1.18
T3	0.820 ab ± 0.005	99.22 b ± 0.77	63.78 b ±1.81
T4	0.777 b ± 0.020	77.41 c ± 0.73	71.58 b ± 1.03
T5	0.769 b ± 0.014	98.86 b ± 0.01	62.32 b ±1.42
T6	0.503 c ± 0.007	32.91 d ± 1.66	87.98 a ± 0.10
T7	0.500 c ± 0.004	30.27 d ± 1.36	90.07 a ± 0.02
T8	0.502 c ± 0.012	30.01 d ± 0.37	88.37 a ± 0.29
T9	0.0000 d ± 0.00	0.04 e ± 0.00	99.99 a ± 0.00

Means with the same letters within columns are not significantly different at *p* ≤ 0.05, according to the Tukey test, and are also presented as mean ± SD (*n* = 4). T1 = water (control); T2 = crude extract of *Garcinia atroviridis*; T3 = nanoemulsion of crude extract (NE4); T4 = nanoemulsion of crude extract (NE7); T5 = aqueous suspension of *T. harzianum* T6 = Dithane M-45^®^ @ 2 g L^−1^; T7 = nanoemulsion of crude extract (NE4) + *T. harzianum*; T8 = nanoemulsion of crude extract (NE7) + *T. harzianum*; T9 = healthy (uninoculated).

**Table 4:** Disease progress rates, area under disease progress curve (AUDPC) and percentage anthracnose disease reduction in chilli fruits inoculated with *C. capsici* and treated with different nanoemulsions either alone or integrated with *T. harzianum*.

Treatment	Disease Progress Rate (Units/Week)	AUDPC (Unit Square)	(%) Reduction
T1	0.9584 a ± 0.0690	342 a ± 3.05	0 e ± 0.00
T2	0.8689 ab ± 0.1100	132.81 b ± 3.39	61.14 d ±2.11
T3	0.7742 b ± 0.1100	104.69 b ± 2.15	67.88 d ± 0.87
T4	0.8281 ab ± 0.0900	76.56 c ± 2.91	76.18 c ± 1.61
T5	0.7271 b ± 0.2100	98.44 b ± 1.82	68.40 d ± 1.65
T6	0.6250 c ± 0.1000	34.38 d ± 2.13	89.27 b ± 2.08
T7	0.5313 d ± 0.1000	26.56 d ± 3.08	91.86 a ± 1.20
T8	0.5985 d ± 0.2000	32.81 d ± 1.57	89.87 b ± 2.94
T9	0.00 e ± 0.0000	0.00 e ± 0.00	100 a ± 0.00

Means with the same letters within columns are not significantly different at *p* ≤ 0.05, according to the Tukey test, and are also presented as mean ± SD (*n* = 4). T1 = water (control); T2 = crude extract of *Garcinia atroviridis*; T3 = nanoemulsion of crude extract (NE4); T4 = nanoemulsion of crude extract (NE7); T5 = aqueous suspension of *T. harzianum* T6 = Dithane M-45^®^ @ 2 g L^−1^; T7 = nanoemulsion of crude extract (NE4) + *T. harzianum*; T8 = nanoemulsion of crude extract (NE7) + *T. harzianum*; T9 = healthy (uninoculated).

**Table 5:** Treatments for evaluating the effectiveness of the integrated use of *G. atroviridis* fruit extract nanoemulsion and *T. harzianum* on anthracnose development in chilli and their effect on total yield.

Treatment	Inoculated with *C. capsici*
T1	Water
T2	Crude extract of *G. atroviridis*
T3	Nanoemulsion 1 (NE4) (250 mL plant^−1^)
T4	Nanoemulsion 2 (NE7) (250 mL plant^−1^)
T5	Aqueous suspension of *T. harzianum* (250 mL plant^−1^)
T6	Dithane M-45^®^ @ 2 g L^−1^
T7	Nanoemulsion 1 (NE4) + aqueous suspension of *T. harzianum* (250 mL plant^−1^) 125 mL each
T8	Nanoemulsion 2 (NE7) + aqueous suspension of *T. harzianum* (250 mL plant^−1^) 125 mL each
T9	Water (without inoculation)

## Data Availability

The original contributions presented in this study are included in the article/Supplementary Materials. Further inquiries can be directed to the corresponding author.

## Supplementary Materials

The following supporting information can be downloaded at: https://www.mdpi.com/article/10.3390/plants15081214/s1, Table S1. Percentage (*w*/*w*) composition of surfactant, oil, water and crude extract; Table S2. Stability and thermostability assessment of selected pre-formulations by centrifugation and storage at different temperatures and storage periods; Table S3. Standard Nutrient Solution used in fertigation system; Figure S1. Pseudo ternary phase diagram of Emereen oil/surfactant/water (Shaded area) L = isotropic, MP = multiphase; Figure S2. (a) Development of Anthracnose symptoms caused by *C. capsici* after 8 weeks of assessment in chilli plants receiving T1, control: The infected plants manifesting necrotic and chlorotic symptoms followed by extensive shoot dieback; (b) T8, Nano-emulsion of crude extract (NE7) + *T. harzianum*; (c) T7, Nano-emulsion of crude extract (NE4) + *T. harzianum*: the plants remained healthy with mild necrotic symptoms on the leaves (Arrow); (d) T6, Dithane M45^®^: marginal scorching of leaves; (e), T3, Nano-emulsion of crude extract (NE7): stem and branches with water-soaked lesions covered with mycelium (arrow); (f) T2, Crude extracts of *G. atroviridis*: plants with leaf spot and excessive chlorosis spots and browning evident in fruits (arrow).
