# Supplementary material for: Management of Chilli Anthracnose Using Garcinia atroviridis Nanoemulsions Integrated with Trichoderma harzianum
Source: Plants (Basel). 2026 Apr 15;15(8):1214. doi: 10.3390/plants15081214 (PMC13119246; doi:10.3390/plants15081214)
Supplement: Supplementary file 1 [file plants-15-01214-s001.zip › plants-4174967-supplementary.pdf]

Supplementary Materials

Table S1. Percentage (*w/w*) composition of surfactant, oil, water and crude extract.

Sample	Tween 80 (surfactant) (w/w)	Emeeren oil (w/w)	Water (w/w)
PF1	25	20	55
PF2	20	40	40
PF3	23	10	67
PF4	20	30	50
PF5	15	35	50
PF6	15	30	55
PF7	5	5	90

Table S2. Stability and thermostability assessment of selected pre-formulations by centrifugation and storage at different temperatures and storage periods.

Pre-formulation	Centrifugation	Temperature 28^{c}c 60 days	Temperature 54^{0}c 14 days	Temperature 4^{0}c 14 days
PF1	/	/	/	X
PF2	/	/	X	X
PF3	/	X	/	X
PF4	/	/	/	X
PF5	/	/	X	X
PF6	/	/	/	X
PF7	/	/	/	X

/- Stable; X- unstable

Table S3. Standard Nutrient Solution used in fertigation system.

Stock A (30 L)		Stock B (30 L)	
**Compound**	**Amount**	**Compound**	**Amount**
Calcium Nitrate	6.34 kg	Potassium Sulfate	3.88 kg
Potassium Nitrate	1.18 kg	Magnesium Sulfate	3.566 kg
Ferrum Chelate	211.3 gm	Manganese Sulfate	26.46 gm
		Boron	38.8 gm
		Copper	8.8 gm
		Ammonium Molybdate	1.0 gm
		Zinc	8.8 gm
		Monopotassium Phosphate	570 gm

Supplementary Figure

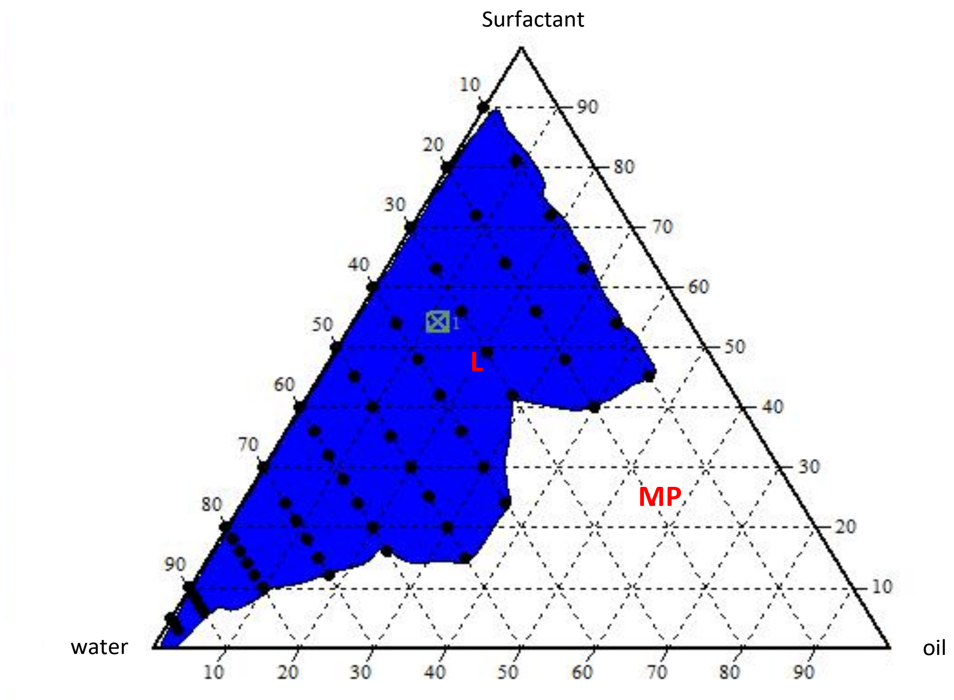

Figure S1. Pseudo ternary phase diagram of Emereen oil/surfactant/water (Shaded area) L = isotropic, MP = multiphase.

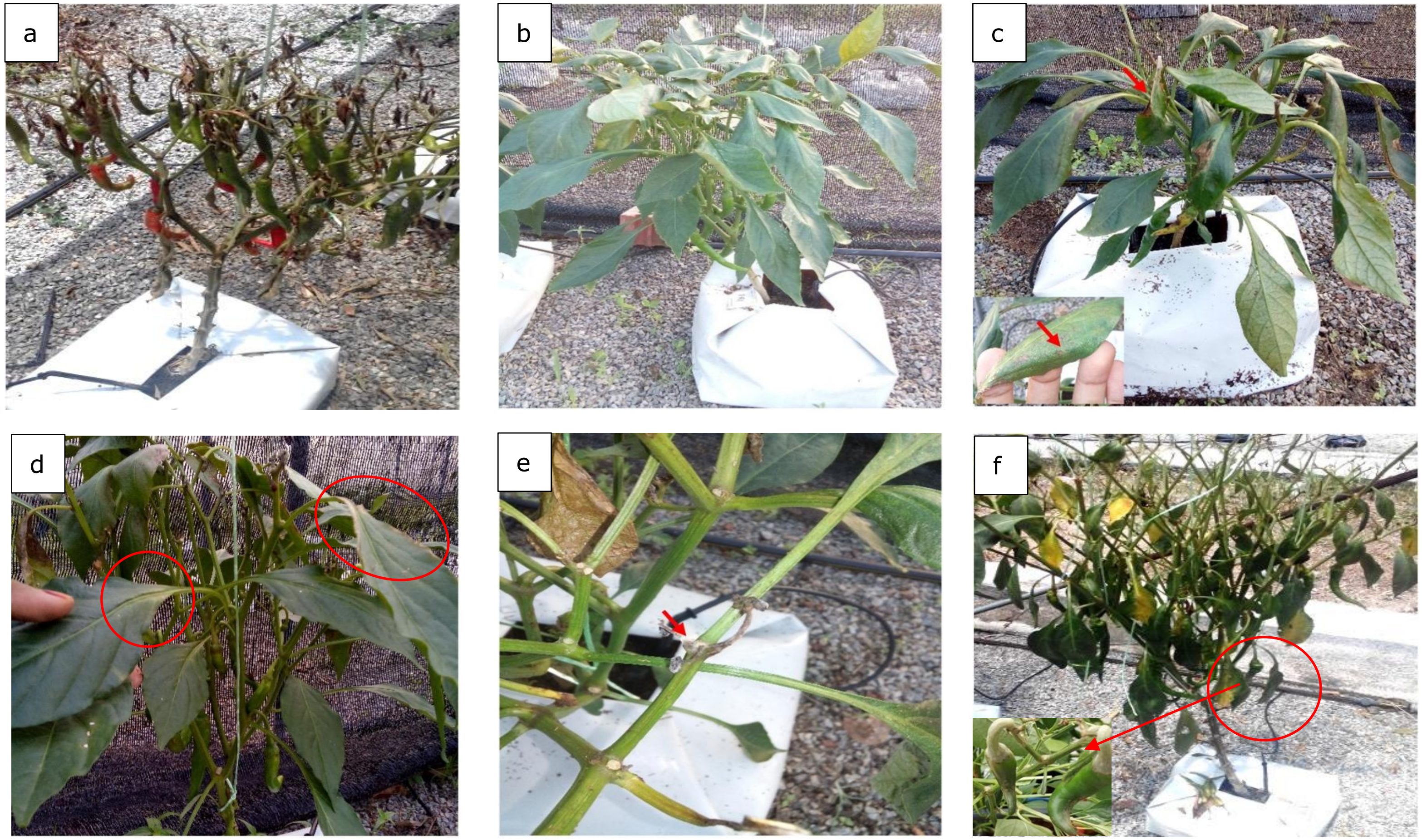

Figure S2. (a) Development of Anthracnose symptoms caused by *C. capsici* after 8 weeks of assessment in chilli plants receiving T1, control: The infected plants manifesting necrotic and chlorotic symptoms followed by extensive shoot dieback; (b) T8, Nano-emulsion of crude extract (NE7) + *T. harzianum*; (c) T7, Nano-emulsion of crude extract (NE4) + *T. harzianum*: the plants remained healthy with mild necrotic symptoms on the leaves (Arrow); (d) T6, Dithane M45® : marginal scorching of leaves; (e), T3, Nano-emulsion of crude extract (NE7): stem and branches with water-soaked lesions covered with mycelium (arrow); (f) T2, Crude extracts of *G. atroviridis*: plants with leaf spot and excessive chlorosis spots and browning evident in fruits (arrow).
